# Association between duration of smoking cessation and depressive symptoms among community-dwelling older adults: a cross-sectional study

**DOI:** 10.1265/ehpm.26-00090

**Published:** 2026-10-03

**Authors:** Kanon Abe, Sho Nakakubo, Takehiko Doi, Shinnosuke Nosaka, Fumio Sakimoto, Soichiro Matsuda, Hiroyuki Shimada

**Affiliations:** 1 Department of Preventive Gerontology, Center for Gerontology and Social Science, National Center for Geriatrics and Gerontology, Obu, Aichi, 474-8511, Japan; 2 Digital Health Research Team, Center for Gerontology and Social Science, National Center for Geriatrics and Gerontology, Obu, Aichi, 474-8511, Japan; 3 Health Innovation Center, Kio University, Nara, 635-0832, Japan; 4 Cognitive Function Research, Aging Research (Partnership field), Nagoya University Graduate School of Medicine, Nagoya, Aichi, 466-8550, Japan; 5 Medical Science Division, Department of Medical Sciences, Graduate School of Medicine, Science and Technology, Shinshu University, Matsumoto, Nagano, 390-8621, Japan

**Keywords:** Smoking, Smoking cessation, Depressive symptoms, Older adults

## Abstract

**Background:**

Smoking is associated with depressive symptoms, and smoking cessation has been associated with improved mental health; however, the specific duration and timing of smoking cessation remain unclear. Persuasive evidence is required to support smoking cessation among older adults with long histories of smoking. Consequently, we aimed to examine the associations among duration of smoking cessation, age at smoking cessation, and depressive symptoms among community-dwelling older adults.

**Methods:**

This cross-sectional study included 20,950 older adults from the National Center for Geriatrics and Gerontology–Study of Geriatric Syndromes. Participants were classified as never, former, or current smokers. Former smokers were further divided into eight groups according to the number of years since smoking cessation. Depressive symptoms were assessed using the 15-item Geriatric Depression Scale, with scores ≥6 defined as depressive symptoms. Multivariable logistic regression was used to estimate adjusted odds ratios (AORs) for depressive symptoms according to smoking status.

**Results:**

Among the 20,142 participants, 6.6%, 32.0%, and 61.4% were current, former, and never smokers, respectively. Overall, 2,389 participants had depressive symptoms. Compared with current smokers, those who had quit smoking for ≥7 years had significantly lower odds of depressive symptoms (AORs = 0.540–0.718). Participants who quit smoking after the age of 65 years also had lower odds of depressive symptoms than current smokers (AOR = 0.765).

**Conclusions:**

Long-term smoking cessation, even later in life, was associated with lower odds of depressive symptoms. These findings highlight the importance of promoting smoking cessation and supporting long-term abstinence among middle-aged and older adults.

**Trial registration:**

Not applicable

**Supplementary information:**

The online version contains supplementary material available at https://doi.org/10.1265/ehpm.26-00090.

## Background

The age-standardized smoking rates were 25.6% among men and 6.9% among women in Japan in 2023 [1]. Smoking significantly increases the risk of severe health problems, including cardiovascular disease, respiratory illnesses, and cancer [2–4]. It is also closely related to mental disorders. The smoking rate and proportion of heavy smokers are higher among individuals with psychiatric diseases than in the general population [5]. Recent research has shown that, compared with never smokers, former smokers have 1.08-fold higher odds of depressive symptoms, whereas current smokers have 1.24-fold higher odds [6].

Although the physical and mental harms of smoking are well established, evidence shows that their detrimental effects are reversible following smoking cessation. For example, the excess risk of pulmonary disease due to smoking can be eliminated within 20 years of cessation [7]. Among light former smokers, the risk of cardiovascular disease significantly decreases within 10 years of cessation [8]. Similar trends have been observed for mental health. Data from the US National Health and Nutrition Examination Survey of adults across all age groups indicate that longer smoking abstinence is associated with fewer depressive symptoms [9].

Despite the well-established benefits of smoking cessation, nicotine addiction remains a major barrier [10]. Moreover, smoking cessation may be more challenging among older adults because of long-standing nicotine dependence, entrenched smoking habits, and underestimation of their susceptibility to smoking-related harm [11]. However, substantial health gains have also been demonstrated among those who quit later in life; for example, quitting at age 65 years is associated with a 23.4% higher probability of surviving for at least one additional year, and cessation at age 75 years still increases survival by approximately 14.2% [12].

Evidence also suggests that smoking cessation may be associated with better mental health outcomes. A recent systematic review and meta-analysis reported that successful smoking cessation was associated with better mental health outcomes, and a large longitudinal cohort study similarly found that smoking cessation was associated with lower levels of common mental health symptoms [13, 14]. However, these studies primarily included middle-aged or mixed-age populations. In contrast, a 1-year longitudinal study reported that smoking cessation after age 55 years was associated with a higher likelihood of depressive symptoms compared with continued smoking [15]. Thus, the association between smoking cessation and depressive symptoms in later life remains unclear, and few studies have specifically focused on community-dwelling older adults. Because late-life depressive symptoms are strongly associated with reduced quality of life and increased mortality, clarifying this association has important clinical and public health implications [16, 17].

Therefore, this study aimed to examine the associations between duration since smoking cessation, age at smoking cessation, and depressive symptoms among community-dwelling older adults.

## Methods

### Study design and participants

This study enrolled participants from the National Center for Geriatrics and Gerontology–Study of Geriatric Syndromes (NCGG-SGS), conducted across several sites in Aichi Prefecture, Japan [18]. The NCGG-SGS is a large community-based cohort study designed to identify the risks of geriatric syndromes associated with aging and to determine effective ways to manage them. We performed a cross-sectional analysis of data from 20,950 older adults who participated in the NCGG-SGS in four municipalities: Tokai, Toyoake, Chita, and Midori Ward of Nagoya. All individuals residing in the survey areas at the time of the study, including those aged ≥60 years in Chita, ≥65 years in Tokai and Toyoake, and ≥70 years in Midori Ward, Nagoya City, were eligible for inclusion. The survey was conducted from September 2017 to August 2024: Tokai (September 2017–June 2018), Toyoake (September 2017–June 2018), Chita (October 2019–March 2021), and Midori Ward, Nagoya City (June 2023–August 2024). Data were collected during face-to-face assessments. Nurses interviewed participants to obtain their medical histories and medication use. Other baseline characteristics, including smoking habits and depressive symptoms, were assessed by well-trained research personnel. Participants were excluded if they met any of the following criteria: certification for long-term care or support under Japan’s public long-term care insurance system (n = 152), disability in basic activities of daily living (n = 43), a history of dementia (n = 45), severe cognitive impairment (Mini-Mental State Examination score <21; n = 381), a history of Parkinson’s disease (n = 54), or missing information required to determine eligibility for these exclusion criteria (n = 88).

### Variables

Participants were classified as never, former, or current smokers. For participants with a history of smoking, we recorded the age at smoking initiation and, for former smokers, the age at smoking cessation. Duration since smoking cessation was defined as the difference between the participant’s current age and age at smoking cessation. Depressive symptoms were assessed using the 15-item Geriatric Depression Scale (GDS-15), with scores ≥6 indicating depressive symptoms [19–21]. This cutoff has been validated for Japanese older adults [22]. Based on previous studies [23, 24], covariates included age, sex, number of prescribed medications, presence of each comorbidities (hypertension, diabetes, stroke, or respiratory disease), equivalized income (<2 million JPY, 2–4 million JPY, or >4 million JPY) [25, 26], living alone, exercise frequency (≥1 day per week or <1 day per week), sleep satisfaction (satisfied or dissatisfied), Mini-Mental State Examination score, and alcohol consumption. Additionally, the municipality (Tokai City, Toyoake City, Chita City, and Midori Ward of Nagoya City) was also included as a covariate to account for potential differences in participant recruitment and population characteristics across study areas.

### Statistical analyses

Missing data were handled as follows. Smoking-related variables (smoking status, duration since smoking cessation, and age at smoking cessation), which were the primary exposure variables, and depressive symptoms (GDS-15 score), which was the outcome variable, were not imputed. Participants with missing data on these variables were excluded from the analyses (n = 8 for smoking-related variables and n = 37 for depressive symptoms). Among the remaining eligible participants, 1,592 had missing data for one or more covariates (number of missing values: prescribed medications, n = 26; hypertension, n = 1; diabetes, n = 9; stroke, n = 3; respiratory disease, n = 7; equivalized income, n = 1,531; number of household members, n = 241; exercise frequency, n = 9; sleep satisfaction, n = 3; alcohol consumption, n = 1). Missing covariate data were addressed using multiple imputation by chained equations. The imputation model included all variables used in the primary analyses. A total of 50 imputed datasets were generated and combined using Rubin’s rules.

Continuous variables were expressed as means (standard deviations [SDs]), and categorical variables as numbers and percentages. Baseline characteristics were compared between participants with and without depressive symptoms using the t-test for continuous variables and the chi-square test for categorical variables. Baseline characteristics across smoking status groups were compared using one-way analysis of variance or chi-square test.

To visualize the association between duration since smoking cessation and depressive symptoms, we modeled it using restricted cubic splines and presented the results as adjusted odds ratios (AORs) with 95% confidence intervals (CIs), with current smokers (0 years since smoking cessation) as the reference [27]. The spline models were adjusted for the covariates described in the Variables section using the multiply imputed datasets. Four knots were utilized, with the outer percentiles set at 0.05 and 0.95.

Moreover, based on a previous study [28], we examined whether the proportion of participants with depressive responses for each GDS-15 item changed with increasing duration since smoking cessation using the Cochran–Armitage trend test to identify the GDS-15 subdomains most closely associated with smoking cessation.

To identify factors associated with depressive symptoms, we performed multivariable logistic regression analyses and estimated AORs with 95% CIs using the multiply imputed datasets. The dependent variable was depressive symptoms (GDS score ≥6). The exposure variable was smoking history. Participants were categorized into 10 groups: current smokers; those who had quit smoking for <4, 4–6, 7–9, 10–12, 13–15, 16–18, 19–21, or ≥22 years; and never smokers. The models were adjusted for the covariates described in the Variables section.

Additionally, to examine whether smoking cessation initiated after entering older adulthood was associated with depressive symptoms, former smokers were categorized according to age at smoking cessation (<65 years or ≥65 years). Age 65 years was selected a priori because it is the conventional threshold for defining older adulthood in public health statistics and epidemiological studies, including national aging statistics in Japan and international indicators of the older population. In addition, smoking cessation after age 65 remains clinically meaningful, as previous studies have shown health benefits even when cessation occurs later in life [12]. We performed a separate multivariable logistic regression analysis in which participants were classified according to smoking history as current smokers, those who had quit smoking before age 65 years, those who had quit smoking at age ≥65 years, and never smokers. Statistical analyses were performed using IBM SPSS Statistics 30 (IBM Japan, Tokyo, Japan) or R version 4.5.0, and p < 0.05 was considered statistically significant.

## Results

### Participants’ characteristics

A total of 20,142 participants (Tokai, n = 5,386; Toyoake, n = 4,831; Chita, n = 6,189; Midori Ward of Nagoya, n = 3,736) were included in the analysis after applying the exclusion criteria (mean age, 74.5 [SD, 6.2] years; 54.1% female; Table 1). The number of participants and age distribution by municipality are presented in Additional File 1.

**Table 1:** Participants’ characteristics according to depressive symptoms

	**All**		**without** **depressive** **symptoms**		**with depressive** **symptoms**		**p value**

	**(N = 20,142)**		**(N = 17,753)**		**(N = 2,389)**		
Age, years							
Mean age (SD), y	74.5 (6.2)		74.4 (6.1)		75.4 (6.6)		<0.001
60–69	4,644	23.1%	4,150	23.4%	494	20.7%	<0.001
70–79	11,068	54.9%	9,842	55.4%	1,226	51.3%	
80–89	4,235	21.0%	3,607	20.3%	628	26.3%	
≥90	195	1.0%	154	0.9%	41	1.7%	
Sex							
Female	10,893	54.1%	9,643	54.3%	1,250	52.3%	0.066
Male	9,249	45.9%	8,110	45.7%	1,139	47.7%	
Mean number of prescribed medications (SD)	3.4 (3.0)		3.2 (2.9)		4.2 (3.4)		<0.001
Missing	36	0.2%	32	0.2%	4	0.2%	
Disease history							
Hypertension	9,606	47.7%	8,388	47.2%	1,218	51.0%	<0.001
Diabetes	2,799	13.9%	2,415	13.6%	384	16.1%	0.001
Stroke	1,111	5.5%	908	5.1%	203	8.5%	<0.001
Respiratory disease	2,572	12.8%	2,205	12.4%	367	15.4%	<0.001
Mean MMSE score (SD), points	27.1 (2.3)		27.2 (2.3)		26.7 (2.5)		<0.001
Equivalized income, JPY							
>2 million	8,583	42.6%	7,341	41.4%	1,242	52.0%	<0.001
2–4 million	7,951	39.5%	7,164	40.4%	787	32.9%	
≥4 million	2,077	10.3%	1,952	11.0%	125	5.2%	
Missing	1,531	7.6%	1,296	7.3%	235	9.8%	
Living alone							
No	16,302	80.9%	14,538	81.9%	1,764	73.8%	<0.001
Yes	3,599	17.9%	3,023	17.0%	576	24.1%	
Missing	241	1.2%	192	1.1%	49	2.1%	
Regular exercise							
≥1 d/wk	15,888	78.9%	14,263	80.3%	1,625	68.0%	<0.001
<1 d/wk	4,245	21.1%	3,482	19.6%	763	31.9%	
Missing	9	0.0%	8	0.0%	1	0.0%	
Sleep satisfaction							
Satisfied	15,989	79.4%	14,511	81.7%	1,478	61.9%	<0.001
Dissatisfied	4,150	20.6%	3,239	18.2%	911	38.1%	
Missing	3	0.0%	3	0.0%	0	0.0%	
Alcohol consumption							
No	11,576	57.5%	10,099	56.9%	1,477	61.8%	<0.001
Yes	8,565	42.5%	7,653	43.1%	912	38.2%	
Missing	1	0.0%	1	0.0%	0	0.0%	
Smoking							
Current	1,327	6.6%	1,098	6.2%	229	9.6%	<0.001
Former	6,445	32.0%	5,649	31.8%	796	33.3%	
Never	12,370	61.4%	11,006	62.0%	1,364	57.1%	

SD, standard deviation; JPY, Japanese yen; MMSE, Mini-Mental State Examination

Depressive symptoms were classified based on the 15-item Geriatric Depression Scale: ≤5, without depressive symptoms; ≥6, with depressive symptoms. Missing values of disease history: Hypertension, n = 1; Diabetes, n = 9; Stroke, n = 3; Respiratory disease, n = 7. Statistical analysis was performed using the t-test or the chi-square test.

Regarding smoking history, 6.6% were current smokers, 32.0% were former smokers, and 61.4% had never smoked. The prevalence of depressive symptoms (GDS score ≥6) was 11.9%. Participants with depressive symptoms were significantly older, had a higher prevalence of a history of diseases, were more likely to have lower income, were more likely to live alone, exercised less frequently, consumed less alcohol, and were more likely to be smokers (all p < 0.05). Among former smokers, the mean duration since smoking cessation was 21.7 years (SD, 16.7). Participants who quit smoking before age 65 years had a substantially longer duration of abstinence (mean, 29.3 years [SD, 13.7]) than those who quit smoking at age ≥65 years (mean, 7.8 years [SD, 5.4]).

Participant characteristics according to smoking status are presented in Additional File 2. Ever smokers were more likely to be male, have no regular exercise habits, and report lower sleep satisfaction than never smokers.

### Association between duration since smoking cessation and depressive symptoms

We employed restricted cubic spline analysis to examine the nonlinear association between duration since smoking cessation and depressive symptoms. After adjusting for age and sex, a longer duration since smoking cessation was associated with a lower probability of depressive symptoms (Fig. 1).

**Fig. 1:**
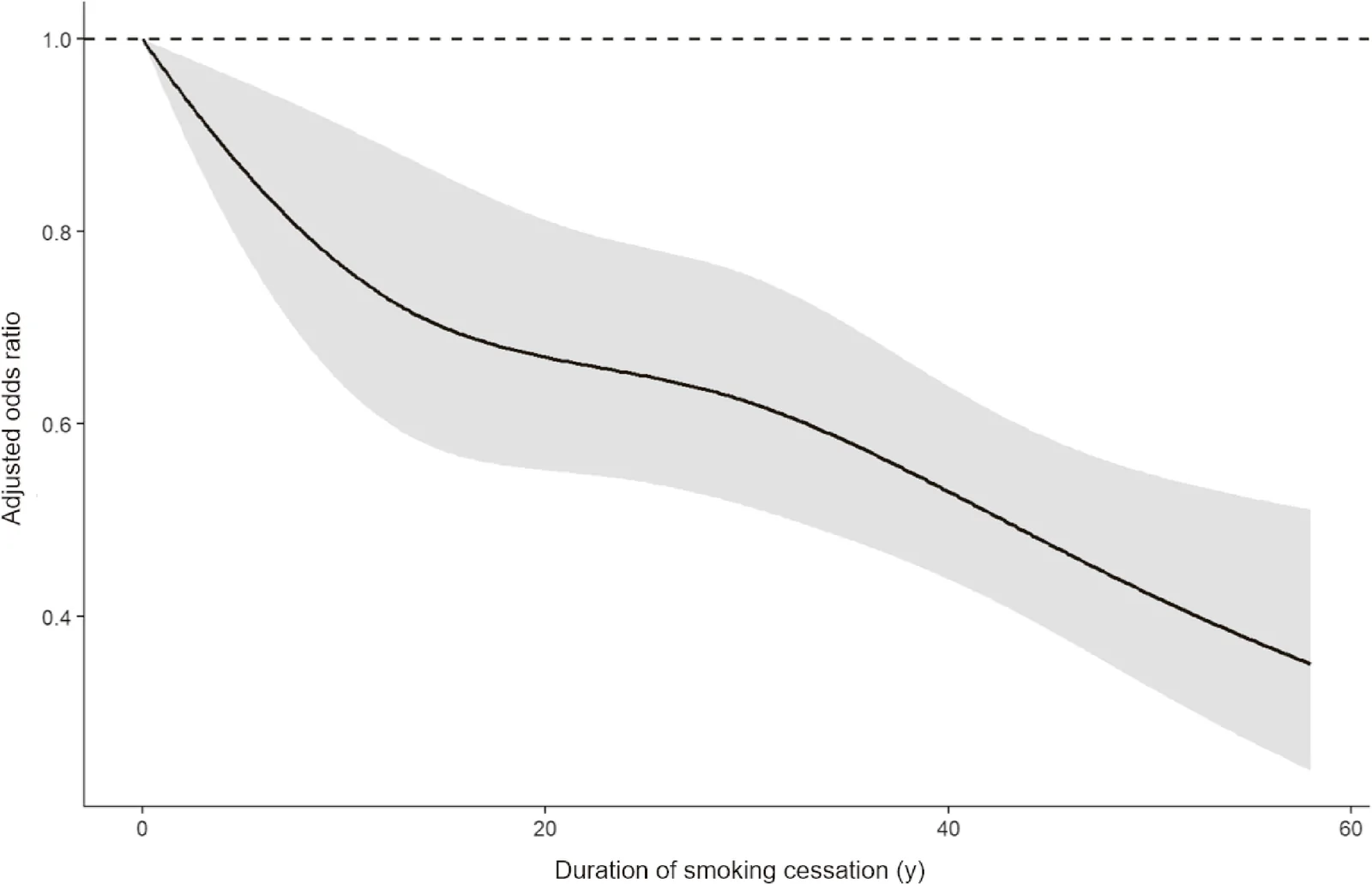
Association between the probability of depressive symptoms and duration of smoking cessation The association between the probability of depressive symptoms and duration of smoking cessation was analyzed using restricted cubic spline analysis, after adjusting for age and sex. Depressive symptoms were defined as a 15-item Geriatric Depression Scale score ≥6. The blue line indicates the probability of depressive symptoms, and the gray area represents the 95% confidence interval.

For each GDS-15 item, we evaluated whether the proportion of depressive responses changed with increasing duration since smoking cessation using the Cochran–Armitage trend test. Items belonging to the Dysphoric Mood (Q1, Q3, Q4, Q5, Q7, Q11, and Q15), Withdrawal–Apathy–Vigor (Q9 and Q13), and Hopelessness (Q8, Q12, and Q14) domains showed a significant trend, indicating that the proportion of depressive symptoms decreased with increasing duration since smoking cessation (Additional File 3). In contrast, items related to anxiety (Q6) and memory problems (Q10) were not significantly associated with duration since smoking cessation.

Using a multivariable-adjusted model, we examined the association between duration since smoking cessation and depressive symptoms. Among former smokers with a duration since smoking cessation of <7 years, the odds of depressive symptoms did not differ significantly from those of current smokers (AOR: <4 years, 0.832; 4–6 years, 0.963; all p > 0.05; Table 2).

**Table 2:** Logistic regression analysis of factors associated with depressive symptoms, including the duration of smoking cessation

**Factors**	**AOR**	**95% CI**	**p value**
Age, y	1.016	1.008–1.025	<0.001
Sex			
Female	Ref		
Male	1.026	0.899–1.171	0.702
Number of prescribed medications, n	1.078	1.062–1.095	<0.001
Hypertension	0.903	0.820–0.995	0.039
Diabetes	0.951	0.837–1.081	0.444
Stroke	1.380	1.165–1.635	<0.001
Respiratory disease	1.074	0.946–1.220	0.268
Equivalized income, JPY			
<2 million	Ref		
2–4 million	0.679	0.615–0.750	<0.001
≥4 million	0.399	0.328–0.485	<0.001
Living alone			
No	Ref		
Yes	1.557	1.397–1.735	<0.001
Regular exercise			
≥1 d/wk	Ref		
<1 d/wk	1.784	1.616–1.969	<0.001
Sleep satisfaction			
Satisfied	Ref		
Dissatisfied	2.906	2.642–3.196	<0.001
MMSE score, points	0.945	0.927–0.964	<0.001
Alcohol consumption			
No	Ref		
Yes	0.850	0.770–0.939	0.001
Smoking			
Current smoker	Ref		
Quit for <4 y	0.832	0.587–1.180	0.302
Quit for 4–6 y	0.963	0.675–1.373	0.834
Quit for 7–9 y	0.701	0.492–0.999	0.049
Quit for 10–12 y	0.636	0.461–0.878	0.006
Quit for 13–15 y	0.618	0.453–0.843	0.002
Quit for 16–18 y	0.718	0.525–0.982	0.038
Quit for 19–21 y	0.619	0.452–0.849	0.003
Quit for >21 y	0.540	0.447–0.651	<0.001
Never smoker	0.519	0.432–0.623	<0.001

AOR, adjusted odds ratio; CI, confidence interval; JPY, Japanese yen; MMSE, Mini-Mental State Examination; Ref, Reference.

Response variable: depressive symptoms were classified based on a 15-item Geriatric Depression Scale score ≥6. Models were additionally adjusted for municipality.

In contrast, participants who had quit smoking for ≥7 years had significantly lower odds of depressive symptoms (AOR: 7–9 years, 0.701; 10–12 years, 0.636; 13–15 years, 0.618; 16–18 years, 0.718; 19–21 years, 0.619; >21 years, 0.540; all p < 0.05).

### Association between the age at smoking cessation and depressive symptoms

We further examined the association between age at smoking cessation and depressive symptoms using a multivariable-adjusted model. Participants were categorized into four groups: never smokers, those who had quit smoking before age 65 years, those who had quit smoking at age ≥65 years, and current smokers. Compared with current smokers, those who had quit smoking before age 65 years had significantly lower odds of depressive symptoms (AOR, 0.587; 95% CI, 0.493–0.698; p < 0.001; Table 3). Furthermore, quitting smoking at age ≥65 years was also associated with significantly lower odds of depressive symptoms (AOR, 0.765; 95% CI, 0.604–0.968; p = 0.026).

**Table 3:** Logistic regression analysis of factors associated with depressive symptoms, including the age at smoking cessation

**Factors**	**AOR**	**95% CI**	**p value**
Age, y	1.013	1.005–1.022	0.002
Sex			
Female	Ref		
Male	1.022	0.895–1.167	0.749
Number of prescribed medications, n	1.079	1.063–1.096	<0.001
Hypertension	0.904	0.821–0.996	0.041
Diabetes	0.951	0.837–1.081	0.444
Stroke	1.385	1.170–1.641	<0.001
Respiratory disease	1.078	0.950–1.224	0.244
Equivalized income, JPY			
<2 million	Ref		
2–4 million	0.678	0.614–0.749	<0.001
≥4 million	0.397	0.327–0.483	<0.001
Living alone			
No	Ref		
Yes	1.564	1.404–1.743	<0.001
Regular exercise			
≥1 d/wk	Ref		
<1 d/wk	1.788	1.620–1.973	<0.001
Sleep satisfaction			
Satisfied	Ref		
Dissatisfied	2.899	2.636–3.188	<0.001
MMSE score, points	0.945	0.927–0.964	<0.001
Alcohol consumption			
No	Ref		
Yes	0.849	0.769–0.938	0.001
Smoking			
Current smoker	Ref		
Quit after the age of 65 years	0.765	0.604–0.968	0.026
Quit before the age of 64 years	0.587	0.493–0.698	<0.001
Never smoker	0.521	0.434–0.627	<0.001

AOR, adjusted odds ratio; CI, confidence interval; JPY, Japanese yen; MMSE, Mini-Mental State Examination; Ref, Reference.

Response variable: depressive symptoms were classified based on a 15-item Geriatric Depression Scale (GDS-15) score ≥6. Models were additionally adjusted for municipality.

In sensitivity analyses excluding sleep satisfaction from the adjustment model, the overall pattern of associations was generally similar to that observed in the primary analysis. However, several estimates were attenuated and no longer reached statistical significance, including those for participants who had quit smoking for 7–9 years, 16–18 years, and those who quit smoking at age ≥65 years (Additional Files 3 and 4).

## Discussion

We found that a longer duration since smoking cessation was associated with a lower prevalence of depressive symptoms among community-dwelling older adults. After adjustment for potential confounders, individuals who had abstained from smoking for ≥7 years had significantly lower odds of depressive symptoms than current smokers. In addition, lower odds were observed among individuals who quit smoking after age 65 years. Despite having substantially shorter durations of abstinence, these individuals also had significantly lower odds of depressive symptoms than current smokers. This finding complements the primary duration-based analysis by suggesting that lower odds of depressive symptoms were observed even among participants who had quit smoking later in life.

Previous studies have reported that smoking is associated with depressive symptoms and other common mental health conditions [6, 13, 14]. Several biological mechanisms have been proposed to explain this association. Nicotine promotes the release of serotonin and dopamine, producing transient pleasure; however, because nicotine is highly addictive and has a short half-life, repeated cycles of nicotine exposure and withdrawal may contribute to mood instability and depressive symptoms [29]. In addition, smoking has been associated with chronic systemic inflammation, which has been implicated in the pathophysiology of depression [30]. However, the mechanisms underlying the association between longer smoking abstinence and a lower prevalence of depressive symptoms remain less well understood. Previous studies have suggested that smoking cessation is associated with neurobiological changes related to nicotine withdrawal and adaptation, as well as gradual reductions in systemic inflammation over time [31, 32]. In this context, the lower prevalence of depressive symptoms observed among long-term former smokers may be attributable to recovery from some of the adverse biological effects associated with chronic smoking exposure.

Moreover, long-term smoking abstinence may be accompanied by improvements in several factors associated with depressive symptoms in older adults. Taste perception has been shown to improve within weeks to months after smoking cessation [33, 34]. Exercise tolerance, respiratory function, and respiratory symptoms such as cough and dyspnea also improve progressively over time [35–37]. Sleep quality also improves [38], and engagement in physical activity tends to increase [39]. These changes may indirectly contribute to better mental health among long-term former smokers. Nevertheless, the mechanisms linking long-term smoking abstinence and depressive symptoms remain uncertain and should be investigated in future longitudinal and mechanistic studies. In addition, in sensitivity analyses excluding sleep satisfaction, several associations were attenuated and no longer statistically significant. This may reflect the complex role of sleep as both a potential confounder and a possible mediator linking smoking cessation and depressive symptoms.

Previous studies examining the association between smoking cessation and cognitive decline have demonstrated the benefits of quitting smoking after approximately 20 years [27]. In contrast, our study found no such plateau. The prevalence of depressive symptoms continued to decrease with a longer duration since smoking cessation. One possible explanation for this pattern is the cumulative effect of multiple physiological and behavioral improvements associated with smoking cessation. Sustained smoking abstinence is accompanied by improvements in taste perception, respiratory function, exercise tolerance, respiratory symptoms, sleep quality, physical activity, and overall health status [33–39]. These improvements are closely associated with mental health and may indirectly contribute to lower odds of depressive symptoms. Thus, the absence of a plateau effect in our findings may reflect continuous positive feedback, representing a virtuous cycle of physical and psychological recovery following smoking cessation.

To further explore which aspects of depressive symptoms were associated with smoking cessation, we conducted a subitem analysis of the GDS-15. Depressive symptoms encompass multiple dimensions, with characteristic affective features including decreased positive affect and increased negative affect. In the context of short-term smoking cessation within 6 months, reduced positive affect, manifesting as anhedonia, may be more strongly associated with smoking cessation success than depressed mood [40]. In the present study, subitem analysis of the GDS-15 revealed that a longer duration since smoking cessation was associated with a lower prevalence of depressive symptoms in the Dysphoric Mood, Withdrawal–Apathy–Vigor, and Hopelessness domains. These results suggest that long-term smoking cessation may be particularly associated with improvements in specific affective dimensions of depressive symptoms among older adults.

This study has some limitations. First, because of its cross-sectional design, a causal association between smoking cessation and depressive symptoms could not be determined. Some individuals smoke to alleviate depressive symptoms by stimulating these neurotransmitters, a concept known as the self-medication hypothesis. Longitudinal studies are required to clarify whether a longer duration since smoking cessation is associated with a lower prevalence of depressive symptoms. In addition, sleep satisfaction and other covariates included in the analyses may have complex temporal relationships with both smoking cessation and depressive symptoms. Therefore, longitudinal studies are needed to clarify the temporal relationships among smoking cessation, depression-related factors, and depressive symptoms. Second, information on smoking behavior, including age at smoking cessation, was obtained through self-report. Therefore, recall bias cannot be ruled out, and actual smoking histories may differ from the reported data. Third, this study was conducted in a single prefecture of Japan. Thus, the generalizability of these findings to other regions or countries may be limited.

Despite these limitations, this study has several strengths. It included a large sample of community-dwelling older adults, which enhanced statistical power and external validity. Data were collected by trained nurses and research staff, ensuring high reliability except for variables potentially affected by recall bias. Moreover, the analyses accounted for key factors closely related to depressive symptoms, such as income and sleep satisfaction, allowing for a more accurate assessment of the association between duration since smoking cessation and depressive symptoms. These strengths support the robustness of our findings and provide valuable insights into smoking cessation as a potentially modifiable factor that may be associated with a lower prevalence of depressive symptoms among older adults.

These findings have important clinical and public health implications. Although earlier smoking cessation remains desirable, our findings suggest that smoking cessation may remain associated with a lower prevalence of depressive symptoms even when initiated after age 65 years. At the same time, the lower prevalence of depressive symptoms observed among individuals with longer durations of abstinence underscores the importance of maintaining long-term smoking cessation. Together, these findings support both early smoking cessation and sustained cessation support throughout middle and older adulthood.

## Conclusions

Among community-dwelling older adults, a longer duration since smoking cessation was associated with a lower prevalence of depressive symptoms. Even after adjustment for multiple covariates, participants who had quit smoking for ≥7 years had a significantly lower prevalence of depressive symptoms than current smokers. Furthermore, those who quit smoking after age 65 years had significantly lower odds of depressive symptoms than current smokers. These findings suggest the importance of both early smoking cessation and long-term support to maintain abstinence. Although smoking cessation should ideally occur as early as possible, our findings also suggest that smoking cessation may remain associated with better mental health even when initiated later in life. Together, these findings support continued efforts to promote smoking cessation and sustained abstinence among middle-aged and older adults.

## Abbreviations

AOR: Adjusted Odds Ratio
CI: Confidence Interval
GDS-15: 15-item Geriatric Depression Scale
MMSE: Mini-Mental State Examination
NCGG-SGS: National Center for Geriatrics and Gerontology – Study of Geriatric Syndromes
SD: Standard Deviation
JPY: Japanese Yen

## Supplementary information

**Additional File 1.** Distribution of participants by 5-year age group and municipality. Additional File 1 presents the number and percentage of participants in each 5-year age group according to municipality. **Additional File 2.** Participant characteristics according to smoking status. Additional File 2 presents the baseline characteristics of participants according to smoking status (never smokers, former smokers, and current smokers). Continuous variables are expressed as means (standard deviations), categorical variables as numbers and percentages, and group differences were assessed using one-way analysis of variance or chi-square tests. **Additional File 3.** Sensitivity analysis of the association between duration since smoking cessation and depressive symptoms without adjustment for sleep satisfaction. Additional File 3 presents the results of a sensitivity analysis corresponding to Table 2, in which sleep satisfaction was excluded from the multivariable logistic regression model. **Additional File 4.** Sensitivity analysis of the association between age at smoking cessation and depressive symptoms without adjustment for sleep satisfaction. Additional File 4 presents the results of a sensitivity analysis corresponding to Table 3, in which sleep satisfaction was excluded from the multivariable logistic regression model. **Additional File 5.** Response rates to each item of the GDS-15 according to smoking status and duration of smoking cessation. Additional File 5 presents the proportion of participants corresponding to each GDS-15 item according to the duration of smoking cessation, along with the results of the Cochran–Armitage trend test; items are classified into the Dysphoric Mood, Withdrawal–Apathy–Vigor, and Hopelessness subdomains.

## Declarations

### Availability of data and materials

Data available on request from authors.
