# Supplementary material for: Association between duration of smoking cessation and depressive symptoms among community-dwelling older adults: a cross-sectional study
Source: Environ Health Prev Med. 2026 Oct 3;31:68. doi: 10.1265/ehpm.26-00090 (PMC13649618; doi:10.1265/ehpm.26-00090)
Supplement: Supplementary file 1 [file ehpm-31-068-s001.docx]

**Additional Files**

**Additional File 1. Distribution of participants by 5-year age group and municipality**

|  |  | All | |  | Tokai | |  | Toyoake | |  | Chita | |  | Midori Ward of Nagoya | |
| --- | --- | --- | --- | --- | --- | --- | --- | --- | --- | --- | --- | --- | --- | --- | --- |
|  |  | (N=20,142) | |  | (N=5,386) | |  | (N=4,831) | |  | (N=6,189) | |  | (N=3,736) | |
| Age, years | |  |  |  |  |  |  |  |  |  |  |  |  |  |  |
|  | Mean age (SD), y | 74.5 (6.2) |  |  | 74.1 (5.6) |  |  | 74.2 (5.6) |  |  | 72.6 (6.5) |  |  | 78.6 (5.3) |  |
|  | 60–64 | 769 | 3.8% |  | 0 | 0.0% |  | 0 | 0.0% |  | 769 | 12.4% |  | 0 | 0.0% |
|  | 65–69 | 3,875 | 19.2% |  | 1,383 | 25.7% |  | 1,207 | 25.0% |  | 1,285 | 20.8% |  | 0 | 0.0% |
|  | 70–74 | 5,892 | 29.3% |  | 1,624 | 30.2% |  | 1,474 | 30.5% |  | 1,770 | 28.6% |  | 1,024 | 27.4% |
|  | 75–79 | 5,176 | 25.7% |  | 1,432 | 26.6% |  | 1,237 | 25.6% |  | 1,451 | 23.4% |  | 1,056 | 28.3% |
|  | 80–84 | 3,207 | 15.9% |  | 712 | 13.2% |  | 695 | 14.4% |  | 693 | 11.2% |  | 1,107 | 29.6% |
|  | 85–89 | 1,028 | 5.1% |  | 200 | 3.7% |  | 190 | 3.9% |  | 192 | 3.1% |  | 446 | 11.9% |
|  | ≥ 90 | 195 | 1.0% |  | 35 | 0.6% |  | 28 | 0.6% |  | 29 | 0.5% |  | 103 | 2.8% |

**Additional File 2. Participant characteristics according to smoking status**

|  |  | All | |  | Current smoker | |  | Former smoker | |  | Never smoker | |  | p value |
| --- | --- | --- | --- | --- | --- | --- | --- | --- | --- | --- | --- | --- | --- | --- |
|  |  | (N=20,142) | |  | (N=1,327) | |  | (N=6,445) | |  | (N=12,370) | |  |  |
| Age, years | |  |  |  |  |  |  |  |  |  |  |  |  |  |
|  | Mean age (SD), y | 74.5 (6.2) |  |  | 71.9 (5.7) |  |  | 74.8 (6.2) |  |  | 74.6 (6.2) |  |  | < 0.001 |
|  | 60–69 | 4,644 | 23.1% |  | 509 | 38.4% |  | 1,380 | 21.4% |  | 2,755 | 22.3% |  | < 0.001 |
|  | 70–79 | 11,068 | 54.9% |  | 674 | 50.8% |  | 3,558 | 55.2% |  | 6,836 | 55.3% |  |  |
|  | 80–89 | 4,235 | 21.0% |  | 140 | 10.6% |  | 1,432 | 22.2% |  | 2,663 | 21.5% |  |  |
|  | ≥ 90 | 195 | 1.0% |  | 4 | 0.3% |  | 75 | 1.2% |  | 116 | 0.9% |  |  |
| Sex | |  |  |  |  |  |  |  |  |  |  |  |  |  |
|  | Female | 10,893 | 54.1% |  | 202 | 15.2% |  | 546 | 8.5% |  | 10,145 | 82.0% |  | < 0.001 |
|  | Male | 9,249 | 45.9% |  | 1,125 | 84.8% |  | 5,899 | 91.5% |  | 2,225 | 18.0% |  |  |
| Mean number of prescribed medications (SD) | | 3.4 (3.0) |  |  | 2.8 (2.8) |  |  | 3.7 (3.2) |  |  | 3.2 (2.9) |  |  | < 0.001 |
|  | Missing | 36 | 0.2% |  | 5 | 0.4% |  | 8 | 0.1% |  | 23 | 0.4% |  |  |
| Diease history | |  |  |  |  |  |  |  |  |  |  |  |  |  |
|  | Hypertension | 9,606 | 47.7% |  | 590 | 44.5% |  | 3,360 | 52.1% |  | 5,656 | 45.7% |  | < 0.001 |
|  | Diabetes | 2,799 | 13.9% |  | 226 | 17.0% |  | 1,181 | 18.3% |  | 1,392 | 11.3% |  | < 0.001 |
|  | Stroke | 1,111 | 5.5% |  | 73 | 5.5% |  | 442 | 6.9% |  | 596 | 4.8% |  | < 0.001 |
|  | Respiratory disease | 2,572 | 12.8% |  | 147 | 11.1% |  | 1,055 | 16.4% |  | 1,370 | 11.1% |  | < 0.001 |
| Mean MMSE score (SD), points | | 27.1 (2.3) |  |  | 27.0 (2.4) |  |  | 26.8 (2.4) |  |  | 27.3 (2.3) |  |  | < 0.001 |
| Equivalized income, JPY | | |  |  |  |  |  |  |  |  |  |  |  |  |
|  | > 2 milion | 8,583 | 42.6% |  | 554 | 41.7% |  | 2,666 | 41.4% |  | 5,363 | 43.4% |  | < 0.001 |
|  | 2–4 milion | 7,951 | 39.5% |  | 533 | 40.2% |  | 2,754 | 42.7% |  | 4,664 | 37.7% |  |  |
|  | ≥ 4 million | 2,077 | 10.3% |  | 170 | 12.8% |  | 690 | 10.7% |  | 1,217 | 9.8% |  |  |
|  | Missing | 1,531 | 7.6% |  | 70 | 5.3% |  | 335 | 5.2% |  | 1,126 | 9.1% |  |  |
| Living alone | |  |  |  |  |  |  |  |  |  |  |  |  |  |
|  | No | 16,302 | 80.9% |  | 221 | 16.7% |  | 916 | 14.2% |  | 2,462 | 19.9% |  | < 0.001 |
|  | Yes | 3,599 | 17.9% |  | 1,092 | 82.3% |  | 5,458 | 84.7% |  | 9,752 | 78.8% |  |  |
|  | Missing | 241 | 1.2% |  | 14 | 1.1% |  | 71 | 1.1% |  | 156 | 1.3% |  |  |
| Regular exercise | |  |  |  |  |  |  |  |  |  |  |  |  |  |
|  | ≥ 1 d/wk | 15,888 | 78.9% |  | 851 | 64.1% |  | 4,929 | 76.5% |  | 10,108 | 81.7% |  | < 0.001 |
|  | < 1 d/wk | 4,245 | 21.1% |  | 474 | 35.7% |  | 1,514 | 23.5% |  | 2,257 | 18.2% |  |  |
|  | Missing | 9 | 0.0% |  | 2 | 0.2% |  | 2 | 0.0% |  | 5 | 0.0% |  |  |
| Sleep satisfaction | |  |  |  |  |  |  |  |  |  |  |  |  |  |
|  | Satisfied | 15,989 | 79.4% |  | 1,114 | 83.9% |  | 5,248 | 81.4% |  | 9,627 | 77.8% |  | < 0.001 |
|  | Dissatisfied | 4,150 | 20.6% |  | 213 | 16.1% |  | 1,195 | 18.5% |  | 2,742 | 22.2% |  |  |
|  | Missing | 3 | 0.0% |  | 0 | 0.0% |  | 2 | 0.0% |  | 1 | 0.0% |  |  |
| Alcohol consumption | |  |  |  |  |  |  |  |  |  |  |  |  |  |
|  | No | 11,576 | 57.5% |  | 489 | 36.9% |  | 2,433 | 37.8% |  | 8,654 | 70.0% |  | < 0.001 |
|  | Yes | 8,565 | 42.5% |  | 838 | 63.1% |  | 4,012 | 62.2% |  | 3,715 | 30.0% |  |  |
|  | Missing | 1 | 0.0% |  | 0 | 0.0% |  | 0 | 0.0% |  | 1 | 0.0% |  |  |
| GDS-15 | |  |  |  |  |  |  |  |  |  |  |  |  |  |
|  | ≤ 5 points | 17,753 | 88.1% |  | 1,098 | 82.7% |  | 5,649 | 87.6% |  | 11,006 | 89.0% |  | < 0.001 |
|  | ≥ 6 points | 2,389 | 11.9% |  | 229 | 17.3% |  | 796 | 12.4% |  | 1,364 | 11.0% |  |  |

SD, standard deviation; JPY, Japanese yen; MMSE, Mini-Mental State Examination

Depressive symptoms were classified based on the 15-item Geriatric Depression Scale: ≤ 5, without depressive symptoms; ≥ 6, with depressive symptoms. Missing values of disease history: Hypertension, n = 1; Diabetes, n = 9; Stroke, n = 3; Respiratory disease, n = 7. Statistical analysis was performed using the one-way analysis of variance or chi-square test.

**Additional File 3. Sensitivity analysis of the association between duration since smoking cessation and depressive symptoms without adjustment for sleep satisfaction**

| Factors | | AOR | 95% CI | | | p value |
| --- | --- | --- | --- | --- | --- | --- |
| Age, y | | 1.009 | 1.000 | – | 1.017 | 0.038 |
| Sex | |  |  |  |  |  |
|  | Female | Ref |  |  |  |  |
|  | Male | 0.952 | 0.835 | – | 1.085 | 0.459 |
| Number of prescribed medications, n | | 1.089 | 1.072 | – | 1.105 | < 0.001 |
| Hypertension | | 0.885 | 0.805 | – | 0.974 | 0.012 |
| Diabetes | | 0.936 | 0.825 | – | 1.061 | 0.301 |
| Stroke | | 1.394 | 1.181 | – | 1.646 | < 0.001 |
| Respiratory disease | | 1.137 | 1.004 | – | 1.289 | 0.044 |
| Equivalized income, JPY | |  |  |  |  |  |
|  | < 2 million | Ref |  |  |  |  |
|  | 2–4 million | 0.692 | 0.627 | – | 0.762 | < 0.001 |
|  | ≥ 4 million | 0.406 | 0.334 | – | 0.492 | < 0.001 |
| Living alone | |  |  |  |  |  |
|  | No | Ref |  |  |  |  |
|  | Yes | 1.559 | 1.402 | – | 1.734 | < 0.001 |
| Regular exercise | |  |  |  |  |  |
|  | ≥ 1 d/wk | Ref |  |  |  |  |
|  | < 1 d/wk | 1.793 | 1.627 | – | 1.975 | < 0.001 |
| MMSE score, points | | 0.952 | 0.934 | – | 0.970 | < 0.001 |
| Alcohol consumption | |  |  |  |  |  |
|  | No | Ref |  |  |  |  |
|  | Yes | 0.848 | 0.769 | – | 0.935 | < 0.001 |
| Smoking | |  |  |  |  |  |
|  | Current smoker | Ref |  |  |  |  |
|  | Quit for < 4 y | 0.896 | 0.636 | – | 1.263 | 0.531 |
|  | Quit for 4–6 y | 0.990 | 0.698 | – | 1.403 | 0.954 |
|  | Quit for 7–9 y | 0.739 | 0.522 | – | 1.046 | 0.088 |
|  | Quit for 10–12 y | 0.669 | 0.487 | – | 0.919 | 0.013 |
|  | Quit for 13–15 y | 0.633 | 0.466 | – | 0.860 | 0.003 |
|  | Quit for 16–18 y | 0.749 | 0.551 | – | 1.019 | 0.066 |
|  | Quit for 19–21 y | 0.689 | 0.506 | – | 0.939 | 0.018 |
|  | Quit for > 21 y | 0.576 | 0.479 | – | 0.692 | < 0.001 |
|  | Never smoker | 0.550 | 0.458 | – | 0.659 | < 0.001 |

AOR, adjusted odds ratio; CI, confidence interval; JPY, Japanese yen; MMSE, Mini-Mental State Examination; Ref, Reference.

Response variable: depressive symptoms were classified based on a 15-item Geriatric Depression Scale (GDS-15) score ≥ 6. Models were additionally adjusted for municipality.

**Additional File 4. Sensitivity analysis of the association between age at smoking cessation and depressive symptoms without adjustment for sleep satisfaction**

| Factors | | AOR | 95% CI | | | p value |
| --- | --- | --- | --- | --- | --- | --- |
| Age, y | | 1.006 | 0.998 | – | 1.014 | 0.153 |
| Sex | |  |  |  |  |  |
|  | Female | Ref |  |  |  |  |
|  | Male | 0.948 | 0.832 | – | 1.081 | 0.428 |
| Number of prescribed medications, n | | 1.089 | 1.073 | – | 1.106 | < 0.001 |
| Hypertension | | 0.886 | 0.806 | – | 0.975 | 0.013 |
| Diabetes | | 0.936 | 0.825 | – | 1.062 | 0.302 |
| Stroke | | 1.397 | 1.184 | – | 1.650 | < 0.001 |
| Respiratory disease | | 1.141 | 1.007 | – | 1.293 | 0.038 |
| Equivalized income, JPY | |  |  |  |  |  |
|  | < 2 million |  |  |  |  |  |
|  | 2–4 million | 0.691 | 0.627 | – | 0.762 | < 0.001 |
|  | ≥ 4 million | 0.404 | 0.333 | – | 0.490 | < 0.001 |
| Living alone | |  |  |  |  |  |
|  | No | Ref |  |  |  |  |
|  | Yes | 1.566 | 1.408 | – | 1.741 | < 0.001 |
| Regular exercise | |  |  |  |  |  |
|  | ≥ 1 d/wk | Ref |  |  |  |  |
|  | < 1 d/wk | 1.794 | 1.628 | – | 1.977 | < 0.001 |
| MMSE score, points | | 0.952 | 0.934 | – | 0.970 | < 0.001 |
| Alcohol consumption | |  |  |  |  |  |
|  | No | Ref |  |  |  |  |
|  | Yes | 0.848 | 0.769 | – | 0.935 | < 0.001 |
| Smoking | |  |  |  |  |  |
|  | Current smoker | Ref |  |  |  |  |
|  | Quit after the age of 65 years | 0.818 | 0.649 | – | 1.032 | 0.090 |
|  | Quit before the age of 64 years | 0.622 | 0.525 | – | 0.738 | < 0.001 |
|  | Never smoker | 0.553 | 0.461 | – | 0.663 | < 0.001 |

AOR, adjusted odds ratio; CI, confidence interval; JPY, Japanese yen; MMSE, Mini-Mental State Examination; Ref, Reference.

Response variable: depressive symptoms were classified based on a 15-item Geriatric Depression Scale (GDS-15) score ≥ 6. Models were additionally adjusted for municipality.

**Additional File 5. Response rates to each item of the GDS-15 according to smoking status and duration of smoking cessation**

|  |  | Current smoker | Past smoker (duration of smoking cessation, y) | | | | | | | | Never smoker | p value |
| --- | --- | --- | --- | --- | --- | --- | --- | --- | --- | --- | --- | --- |
| GDS-15 dimensions | GDS-15 items |  | < 4 | 4–6 | 7–9 | 10–12 | 13–15 | 16–18 | 19–21 | > 21 |  |  |
| Dysphoric Mood | Q1 | 13.0% | 15.1% | 12.6% | 10.9% | 8.2% | 11.2% | 8.6% | 10.1% | 8.0% | 7.9% | < 0.001 |
|  | Q3 | 9.9% | 9.7% | 12.6% | 8.8% | 10.4% | 8.9% | 5.8% | 11.0% | 7.3% | 7.3% | < 0.001 |
|  | Q4 | 18.1% | 19.5% | 17.3% | 19.6% | 14.5% | 16.8% | 15.7% | 14.2% | 13.5% | 11.0% | < 0.001 |
|  | Q5 | 5.3% | 5.4% | 5.8% | 4.1% | 2.8% | 5.3% | 4.5% | 3.0% | 3.1% | 4.1% | 0.022 |
|  | Q7 | 17.5% | 15.1% | 14.8% | 14.3% | 11.9% | 14.6% | 12.7% | 11.6% | 9.8% | 8.7% | < 0.001 |
|  | Q11 | 11.8% | 12.4% | 7.9% | 12.6% | 10.8% | 12.6% | 12.1% | 11.4% | 8.0% | 7.0% | < 0.001 |
|  | Q15 | 20.0% | 14.1% | 18.4% | 18.7% | 17.3% | 17.4% | 16.8% | 21.4% | 16.4% | 16.1% | < 0.001 |
|  |  |  |  |  |  |  |  |  |  |  |  |  |
| Withdrawal–Apathy–Vigor | Q2 | 21.6% | 28.5% | 24.5% | 24.6% | 25.8% | 21.3% | 25.0% | 24.7% | 22.6% | 21.7% | 0.066 |
|  | Q9 | 37.0% | 40.9% | 35.7% | 38.9% | 37.7% | 36.3% | 35.8% | 32.1% | 32.0% | 41.8% | < 0.001 |
|  | Q13 | 47.6% | 53.4% | 47.7% | 44.4% | 45.0% | 44.2% | 46.6% | 43.8% | 39.3% | 43.2% | < 0.001 |
|  |  |  |  |  |  |  |  |  |  |  |  |  |
| Anxiety | Q6 | 11.9% | 11.7% | 7.9% | 12.4% | 10.4% | 12.1% | 11.9% | 11.3% | 8.0% | 6.7% | 0.168 |
|  |  |  |  |  |  |  |  |  |  |  |  |  |
| Hopelessness | Q8 | 24.3% | 26.5% | 26.7% | 23.7% | 24.0% | 21.7% | 26.3% | 21.8% | 19.9% | 20.5% | < 0.001 |
|  | Q12 | 6.0% | 6.0% | 6.5% | 5.0% | 4.8% | 5.1% | 5.4% | 5.1% | 4.3% | 3.1% | < 0.001 |
|  | Q14 | 13.3% | 11.7% | 14.1% | 8.8% | 11.0% | 9.9% | 10.6% | 9.5% | 8.2% | 7.8% | < 0.001 |
|  |  |  |  |  |  |  |  |  |  |  |  |  |
| Memory problems | Q10 | 29.4% | 26.5% | 32.5% | 31.6% | 28.6% | 27.4% | 32.1% | 28.1% | 29.2% | 29.2% | 0.815 |

The figure shows the proportion of depressive responses for each GDS-15 item. For Items Q1, Q5, Q7, Q11, and Q13, depressive responses correspond to "No"; for Items Q2, Q3, Q4, Q6, Q8, Q9, Q10, Q12, Q14, and Q15, depressive responses correspond to "Yes."

To identify the specific dimensions of depressive symptoms more closely associated with smoking cessation, we evaluated whether the proportion of depressive responses for each 15-item Geriatric Depression Scale (GDS-15) item increased with a longer duration of smoking cessation, using the Cochran–Armitage trend test.

GDS-15 dimensions: (1) Dysphoric Mood, items Q1, Q3, Q4, Q5, Q7, Q11, and Q15; (2) Withdrawal–Apathy–Vigor, items Q2, Q9, and Q13; (3) Anxiety, item Q6; (4) Hopelessness, items Q8, Q12, and Q14; (5) Memory problems, item Q10.
